# In-Situ One-Step Hydrothermal Synthesis of LiTi_2_(PO_4_)_3_@rGO Anode for High Performance Lithium-Ion Batteries

**DOI:** 10.3390/ma18061329

**Published:** 2025-03-17

**Authors:** Otmane Zoubir, Abdelfettah Lallaoui, M’hamed Oubla, Alvaro Y. Tesio, Alvaro Caballero, Zineb Edfouf

**Affiliations:** 1 Materials and Nanomaterial for Photovoltaics and Electrochemical Storage (MANAPSE), Faculty of Sciences, Mohammed V University in Rabat, Morocco; m.oubla@um5r.ac.ma (M.O.); z.edfouf@um5r.ac.ma (Z.E.); 2 Moroccan Foundation for Advanced Science Innovation and Research (MAScIR), UM6P, Hay Moulay Rachid, CCI, Ben Guerir 43150, Morocco; a.lallaoui@univ-pau.fr; 3 Centro de Investigación y Desarrollo en Materiales Avanzados y Almacenamiento de Energía de Jujuy (CIDMEJu), Centro de Desarrollo Tecnológico General Manuel Savio, Palpalá 4612, Jujuy, Argentina; atesio@cidmeju.unju.edu.ar; 4 Dpto. Química Inorgánica, Instituto Químico para la Energía y el Medioambiente, Universidad de Córdoba, Campus de Rabanales, 14014 Córdoba, Spain

**Keywords:** Li-ion batteries, anode material, NASICON structure, hydrothermal, in situ synthesis

## Abstract

The sodium super ionic conductor (NASICON) structured LiTi_2_(PO_4_)_3_ (LTP) has been developed as electrode material for Li-ion batteries (LIBs) with promising electrochemical performance, owing to its outstanding structural stability and rapid lithium-ion diffusion. Nevertheless, challenges still exist, especially the rapid capacity fading caused by the low electronic conductivity of LTP-NASICON material. Recently, the hydrothermal method has emerged as an important technique for the production of diverse nano-electrode materials due to its low preparation temperature, high phase purity, and well-controlled morphology and crystallinity. Herein, we report, for the first time at low-moderate temperatures, an advanced hydrothermal synthesis of LTP-coated reduced graphene oxide (LTP@rGO) particles that includes the growth of LTP particles while simultaneously coating them with rGO material. The LTP offers a discharge specific capacity of 84 mAh/g, while the LTP@rGO delivers a discharge capacity of 147 mAh/g, both with a coulombic efficiency of 99.5% after 100 cycles at a 1C rate.

## 1. Introduction

Recently, LIBs have received considerable interest owing to their intriguing properties such as high energy density and a long cycle life. They play key roles in diverse applications, ranging from micro electronic devices to electric vehicles and aerospace products [1,2,3,4]. To date, a wide range of electrode materials have been thoroughly investigated to develop powerful batteries [5]. Particularly, anode materials are considered as a critical component in determining the safety and cycling life of LIBs [6,7]. They can be divided into three types based on their reaction mechanism, which includes intercalation (Ti-based oxides e.g., Li_4_Ti_5_O_12_) [8,9,10,11], conversion (oxides e.g., Fe_2_O_3_) [12], and the alloying mechanism (e.g., Li-Si) [13]. However, these materials fall short of meeting the requirement of practical application due to their intrinsic problems including a low Li^+^ diffusion coefficient, poor electronic conductivity, dendrite formation, and volume expansion.

Polyanion-based compounds are considered potential candidates for electrode materials in rechargeable LIBs due to their superior structural stability compared to other oxides [14]. This could be exemplified by the increasingly widespread adoption of the phospho-olivine LiFePO_4_ cathodes [15,16]. NASICON-type materials can be represented as AM_2_(PO_4_)_3_ (A = K, Na, Li; M = Zr, Ge, Ti) within the rhombohedral crystal structure with $R\bar{3}c$ as the space group. The fundamental framework of NASICON-structured materials consists of a rigid [M_2_P_3_O_12_]^−^ skeleton, where three PO_4_ tetrahedra and two MO_6_ octahedra are interconnected by sharing corner oxygen atoms, forming lantern units [17].

In particular, the NASICON-structured LTP material has attracted global interest as a potential electrode material for LIBs [18,19]. It offers significant advantages, including a remarkable structural stability, high ionic conductivity, and fast Li^+^ diffusion [20,21]. However, a major challenge is the rapid capacity fading of LTP anodes, which is due to their low electronic conductivity impeding their practical applications. Despite these advantages, the pure LTP electrode exhibits poor capacity performance, limiting its use in large-scale applications. Therefore, enormous research efforts have been devoted to overcoming this drawback such as nanostructuration, cationic doping, and surface coating. In this regard, various synthesis techniques have been employed to obtain LTP/carbon composites (LTP@C), including co-precipitation [22] and sol-gel [23]. However, these methods often result in agglomerated particles with irregular morphology, hindering their electrochemical performances. Recently, the hydrothermal method was proposed as a green and cost-effective synthetic procedure to prepare various nano-electrode materials for LIBs due to its simplicity and the fact that it can provide high crystallinity powders [24]. Moreover, an aqueous-based synthesis approach entailing the use of autoclave reactors can be easily scaled up to a large-quantity production.

Lui et al. [23] first synthesized LiTi_2_(PO_4_)_3_ via a sol-gel method, followed by a ball milling step with acetylene black to obtain an LiTi_2_(PO_4_)_3_@C nanocomposite. Their findings revealed that the LiTi_2_(PO_4_)_3_@C achieved an initial specific capacity of 140.7 mAh/g at 0.1 C and maintained a capacity retention of 75% after 100 cycles. Roh et al. [25] used a straightforward microwave-assisted one-pot method to produce LiTi_2_(PO_4_)_3_@rGO with a specific capacity of 138 mAh/g at a 0.1C rate, and a high-capacity retention up to 93.2% over 100 cycles. Huang et al. [26] prepared carbon-coated LiTi_2_(PO_4_)_3_ nanoporous microplates (LTP/C MPs) using ethylenediamine as the carbon source. The carbon coating significantly enhanced the electrochemical performance of the material, reaching a capacity of 121 mAh/g at 0.2 C with a high-capacity retention of 94.2% after 100 cycles.

In this work, we report a simple one-step hydrothermal synthesis of LTP@rGO, which combines the in situ reduction of graphene oxide (GO) to rGO material with the simultaneous growth of LTP particles. The LTP@rGO prepared at a temperature as low as 250 °C exhibited a cubic morphology. rGO-embedded high crystalline LTP particles were shown to improve the electronic conductivity. Consequently, the obtained LTP@rGO anode exhibits superior electrochemical performance in comparison to its pristine counterpart, achieved through a simple, cost-effective synthesis method involving fewer steps and lower temperatures compared to the complex, multi-step synthesis procedures previously reported.

## 2. Materials and Methods

### 2.1. Synthesis of LiTi_2_(PO_4_)_3_@rGO

The hydrothermal synthesis was carried out in a Teflon-lined autoclave to obtain the LiTi_2_(PO_4_)_3_ and LiTi_2_(PO_4_)_3_@rGO. Titanium powder Ti (99.5%, Kosh-Light Laboratories, Haverhill, UK), lithium hydroxide monohydrate LiOH.H_2_O (99%, LobaChemie, Mumbai, India), and phosphoric acid H_3_PO_4_ (85%, Sigma-Aldrich, St. Louis, MO, USA) precursors were mixed in a molar ratio of 1:15:45, respectively, to prepare the pristine LTP. The preparation of LTP@rGO consists of replacing the appropriate amount of deionized water (6 mL) with a GO solution (details in Supplementary Material). The GO solution, containing 5 wt% of GO relative to LTP powder, was first sonicated for 30 min to ensure homogenization before being incorporated into the precursor mixture. Subsequently, the resulting mixtures were placed in a Teflon-lined autoclave and heated at 250 °C for 72 h, followed by natural cooling to room temperature (RT). Finally, the LTP and LTP@rGO powders were recovered by filtering and drying at RT without any further calcination steps.

### 2.2. Structural and Morphological Characterizations

X-ray diffraction (XRD) patterns were obtained with a Bruker D8 Discover X-ray diffractometer (Bruker AXS GmbH, Karlsruhe, Germany) with a Cu Kα source (1.5406 Å) operating at 40 kV over a 2ϴ range between 10° and 70°, a 0.04° step size and 1.05 s per step. The crystallite size was evaluated from the Lorentzian contribution to the “Thompson-Cox-Hastings pseudo-Voigt” profile-shape function after considering the instrumental resolution [27]. The Rietveld method was employed for structural refinement using a FullProf program (version 7.40). The TGA (Mettler Toledo-TGA/DSC, Greifensee, Switzerland) was used to assess the thermal behavior as well as the content of the carbon in LTP@rGO under an oxygen atmosphere at a heating rate of 3 °C/min from 25 °C to 800 °C. Scanning electron microscopy (SEM) was employed to assess the samples using (SEM, JEOL JSM–IT 100, JEOL Ltd, Tokyo, Japan, operating at 15 kV). Transmission electron microscopy (TEM) was performed using a Talos F200S microscope (Thermo Fisher Scientific, Waltham, MA, USA) operated at 200 kV. The textural properties were determined using a Micrometrics TriStar II Plus (Micromeritics, Norcross, GA, USA) with nitrogen as an adsorbent, utilizing the Brunauer–Emmett–Teller (BET) method. X-ray photoelectron spectroscopy (XPS) spectra were acquired using a spec “PHOIBOS 150 MCD” spectrometer (SPECS Surface Nano Analysis GmbH, Berlin, Germany), equipped with monochromatic Mg K_α_ radiation and a multichannel detector. All spectra were analyzed with a CasaXPS program, version 2.3.26, to Gaussian–Lorentzian curves. The infrared spectra were recorded with a Bruker Platinum-ATR type spectrometer (Bruker Corporation, Billerica, MA, USA) operating in transmittance mode, with a spectral range from 400 to 4000 cm^−1^. The Raman spectra were recorded with a Renishaw 2000 type device, using a 532 nm laser beam in an ambient atmosphere.

### 2.3. Electrochemical Measurements

The electrode materials were tested electrochemically by assembling coin cells. To prepare the electrodes, 75 wt% of active material, 15 wt% of carbon black and 10 wt% of carboxymethylcelluloses (CMC) binder were mixed in distilled water to create a homogeneous slurry. Then, the slurry was coated onto a Cu foil using a doctor blade (20 μm). The prepared electrodes were dried overnight under vacuum at 120 °C. The final mass loading of the active material is 0.9 ± 0.1 mg/cm^2^. Afterwards, the cells were assembled in a glove box under an argon atmosphere, with lithium foil as a counter-electrode. The electrolyte consisted of 1 M LiPF_6_ dissolved in a 1:1 volume mixture of ethylene carbonate (EC) and diethylene carbonate (DMC). A fiberglass membrane (Whatman 540) was then used as a separator and placed between the counter-electrode and the anode. The cyclic voltammetry (CV) and the Galvanostatic charge/discharge tests were conducted on a Biologic VMP3 potentiostat–galvanostat at RT and electrochemical impedance (EIS) was conducted on a Biologic BCS-805 battery cycler series. CV was carried out at scan rates of 0.01 mV/s across a potential range of 2.5–0.1 V vs. Li^+^/Li. EIS measurements were performed at RT using an excitation potential of 100 mV over a frequency range from 10 KHz to 10 mHz.

## 3. Results and Discussion

### 3.1. Materials Characterization

**Figure 1:**
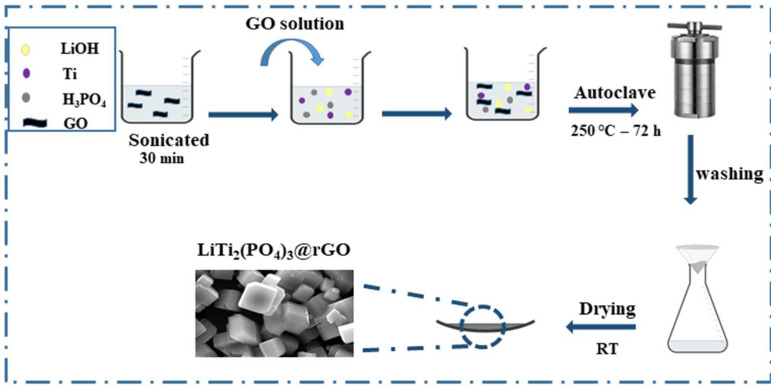
Illustration of the one-step hydrothermal synthesis of LiTi_2_(PO_4_)_3_@rGO.

Figure 1 depicts a schematic of the hydrothermal synthesis process for the LTP and LTP@ rGO composite. Notably, pristine LTP was obtained through a one-step hydrothermal method at a low to moderate temperature and with fewer steps compared to alternative synthesis techniques (e.g., sol-gel or co-precipitation), which typically require multiple steps and high temperatures. Furthermore, our modified synthesis process enables the in situ reduction of GO to rGO during the LTP particle growth, resulting in a uniform distribution of LTP particles on the surface of rGO, making this a more economically viable technique.

**Figure 2:**
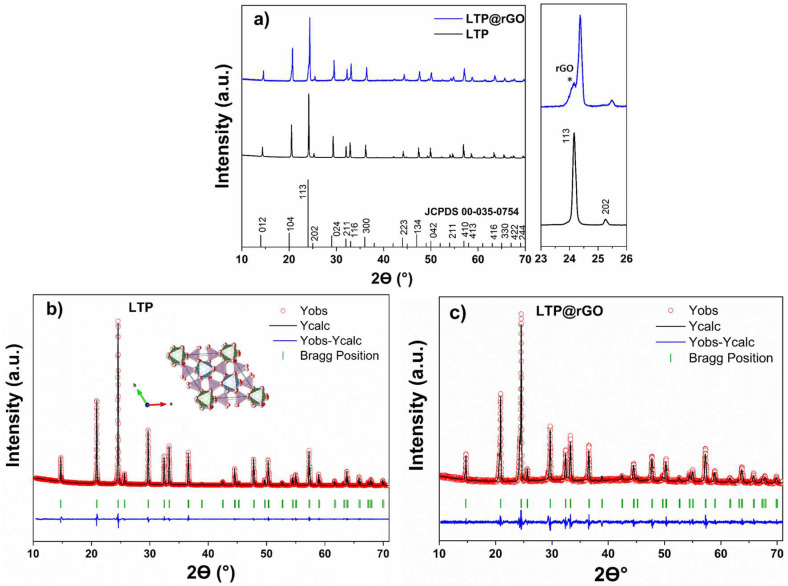
(**a**) XRD patterns of LTP and LTP@rGO (* indicates the presence of rGO), profile matching of (**b**) LTP, (**c**) LTP@rGO.

The XRD pattern of the synthesized GO (Figure S1) shows a wide diffraction peak at 2Ɵ = 10°, confirming the oxidation of graphite into graphene oxide (Figure S2). The XRD pattern (Figure 2a) demonstrates the successful hydrothermal synthesis of the LiTi_2_(PO_4_)_3_ and LiTi_2_(PO_4_)_3_@rGO. The sharp peak intensities of the LTP spectra indicate that the LiTi_2_(PO_4_)_3_ powder is well crystallized. A Rietveld refinement of LTP was performed (Figure S3). The peak shapes of the LTP diagram were fitted using a pseudo-Voigt function and the NASICON-like phosphate structure with 00-035-0754 JCPDS card as a starting model, and $R\bar{3}C$ as the space group. The results (Table S1) show a good agreement between the observed and the calculated diffraction profiles, with good reliability refinement factors; this confirms that the sample is a single phase, and all diffraction peaks are indexed in the standard model. In contrast, the LTP@rGO (Figure 2a) shows a broad reflection between 2Ɵ = 24–25°, corresponding to the formation of rGO flakes [28]. The reduction of GO to rGO during the synthesis process was confirmed by a separate hydrothermal treatment of GO under the same conditions; a broad peak at 24° was observed, suggesting the reduction of GO to rGO (Figure S4). A less intense peak at 44° was also observed in rGO. In addition, a comparison of the lattice parameters (Table 1) was conducted using the refinement results obtained from the profile matching (Figure 2b,c). It is worth noting that the lattice parameters of LTP are quasi-invariant with the rGO coating, indicating that rGO does not induce any significant structural modifications. As determined from peak width analysis, the crystallite size decreased from 87 nm to about 58 nm (Figure S5), suggesting that the rGO could constrain the growth of LTP crystallites.

**Figure 3:**
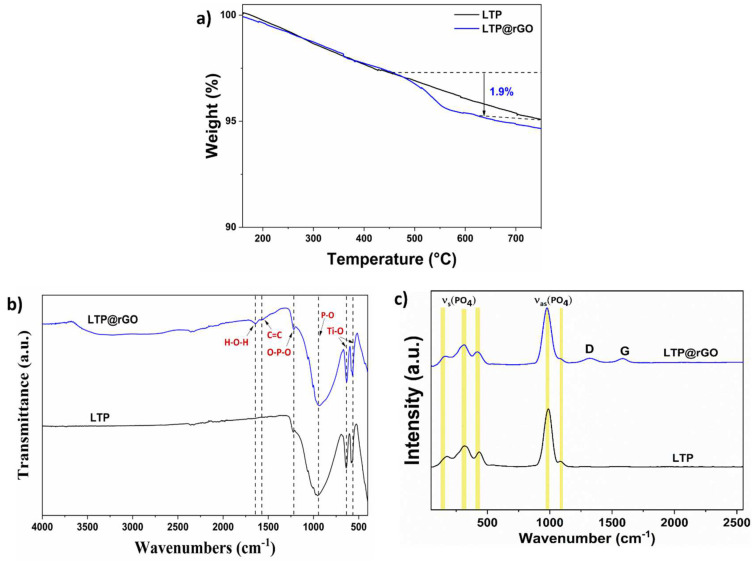
(**a**) TGA curve of LTP and LTP@rGO, (**b**) Infrared spectra of the LTP and LTP@rGO, (**c**) Raman spectra of LTP and LTP@rGO.

The TGA was used to evaluate the amount of the carbon content as well as the thermal stability within the composite. The powder sample was heated from RT up to 700 °C at a rate of 3 °C/min in an O_2_ atmosphere. As shown in Figure 3a, the gradual weight loss between 450 °C and 650 °C can be ascribed to the decomposition of rGO as confirmed in Figure S6. Therefore, the carbon content in the LTP is evaluated to be about 1.9%. The CHNS elemental analysis (Table S2) confirmed the amount around (1.3%) of the incorporated rGO within the LTP. These results also show that no decomposition of LTP was observed in the studied temperature range, which demonstrates excellent thermal stability of the basic crystalline structure.

The FTIR measurement (Figure 3b) was performed to confirm the polyanionic units of the LTP. As can be seen from the FTIR spectra, clear transmission peaks at 575, 639, 980, and 1225 cm^−1^ appear in the sample. The low-frequency part of the spectrum shows moderate absorption peaks at 575 and 639 cm^−1^ that can be assigned to the Ti-O stretching vibration of the TiO_6_ octahedron overlapped with the bending vibration of PO_4_ tetrahedra [29]. The strong peak at 980 cm^−1^ is ascribed to the asymmetric and symmetric stretching vibrations of P-O bands in PO_4_ units while the band at 1225 cm^−1^ corresponds to the bending vibrations of O-P-O in PO_4_ units [23,24]. For LTP@rGO, it approximately features all characteristic peaks of rGO, including a stretching vibration at 570 cm^−1^ of the C=C backbone and an OH vibration at 3400 cm^−1^ of the tertiary C-OH. The lack of the carboxylic C=O group at 1732 cm^−1^ indicates the successful reduction of GO to rGO (Figure S7). A Raman spectra was performed to confirm the presence of rGO in the composite by detecting the associated carbon vibrational bonds.

Figure 3c illustrates the Raman spectra of LTP and LTP@rGO. Clear Raman bands at 148, 311, 438, 991, and 1090 cm^−1^ appear in the sample obtained by hydrothermal synthesis, which are characteristic of triphosphates. The band located at 444 cm^−1^ corresponds to the symmetrical bending vibration of PO_4_ [30], whereas the band at 272 cm^−1^ is assigned to the translational vibration of Ti^4+^ ions [31]. The bands at 150 and 311 cm^−1^ are assigned to modes dominated by PO_4_ motions. The asymmetrical modes of PO_4_ are represented by the bands at 991 cm^−1^ and 1090 cm^−1^, which occur at higher wavenumbers compared to their symmetrical counterparts. The Raman spectra also showed distinct D and G band peaks at 1318 and 1589 cm^−1^, confirming the successful synthesis of the carbonaceous composite. The I_D_/I_G_ ratio for LTP@rGO is about 1.01, indicating that the composite has a high graphitization degree, which could result in good electrical conductivity during the electrochemical processes.

Figure S8 shows the N_2_ adsorption–desorption isotherm of the LTP and LTP@rGO samples. According to the Brunaur–Deming–Teller (BDDT) classification, the LTP isotherms show limited N_2_ uptake, exclusively typical of non-porous or very low-porosity materials. In contrast, LTP@rGO isotherms exhibit a type-IV adsorption with H3-type hysteresis loops at high relative pressure regions (p/p^0^) of 0.9–1, indicating a mesoporous structure. The BET surface analysis shows a total surface area of 16 m^2^ g^−1^ for the LTP@rGO material, which is higher than that of the pristine LTP material (4 m^2^ g^−1^).

**Figure 4:**
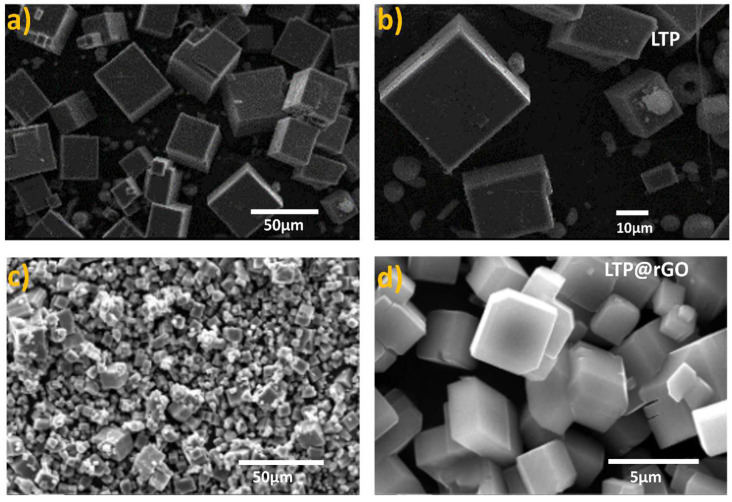
Scanning electron microscopy images of (**a**,**b**) the pristine LTP, (**c**,**d**) the LTP@rGO.

An SEM analysis was used to assess the morphology of the samples. According to the SEM images (Figure 4a,b), the LTP exhibited cubic shapes with well-defined edges, various particle sizes, and smooth surfaces. The average grain size is about 20 µm (Figure S9). Our results agree with the findings of other studies [32,33], in which the morphology of LiTi_2_(PO_4_) prepared by the hydrothermal method was basically rectangular or cubic [34,35]. On the other hand, the SEM images (Figure 4c,d) of LTP@rGO suggest that rGO coating facilitates the reduction in grain size and leads to a smoother surface of the crystals. This behavior can be attributed to the ability of rGO to act as a physical barrier, preventing the coalescence of grains and thereby limiting their growth. As a result, the average particle size was estimated to be around 7 µm (Figure S9).

**Figure 5:**
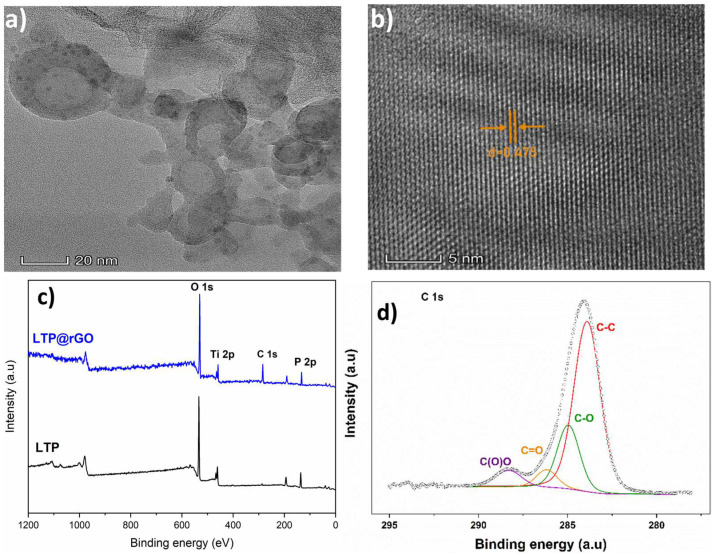
(**a**) TEM image of LTP@rGO, (**b**) HR-TEM image of LTP@rGO, (**c**) survey XPS spectra of LTP and LTP@rGO, (**d**) deconvoluted C 1s peaks of LTP@rGO.

The TEM images (Figure 5a) further show that LTP particles are well dispersed and wrapped by rGO, demonstrating the effectiveness of the in situ coating process. The well-crystallized LTP within the composite is confirmed by the lattice fringes visible in the HR-TEM image (Figure 5b), which agree well with the XRD findings. The lattice parameter of the LTP is 0.475 nm. This aligns with the spacing of the (104) lattice plane determined from the diffraction angle using Bragg’s law. The LTP and LTP@rGO materials were further investigated by XPS and the spectrum was examined using the Gaussian fitting method. As demonstrated in XPS survey spectra (Figure 5c), the materials are composed of Ti, P, O, and C. The binding energies for LTP for Ti 2p_1/2_ and Ti 2p_3/2_ (Figure S10) appear at 465.4 eV and 459.7 eV, respectively, while those for LTP@rGO are observed at 465.12 eV and 459.30 eV, respectively. These results confirm that the Ti^4+^ oxidation state remains unchanged in both LTP and LTP@rGO. The examination of the C 1s region (Figure 5d) reveals the extent of the GO reduction by showing four components, representing the carbon functional groups: C-C (283.89 eV), C-O (284.95 eV), C=O (286.17 eV), and O-C=O (288.30 eV). The results collectively demonstrate the successful synthesis of LTP@rGO with a high degree of GO reduction, thereby enhancing electronic conductivity through the rGO network.

### 3.2. Electrochemical Characterization

**Figure 6:**
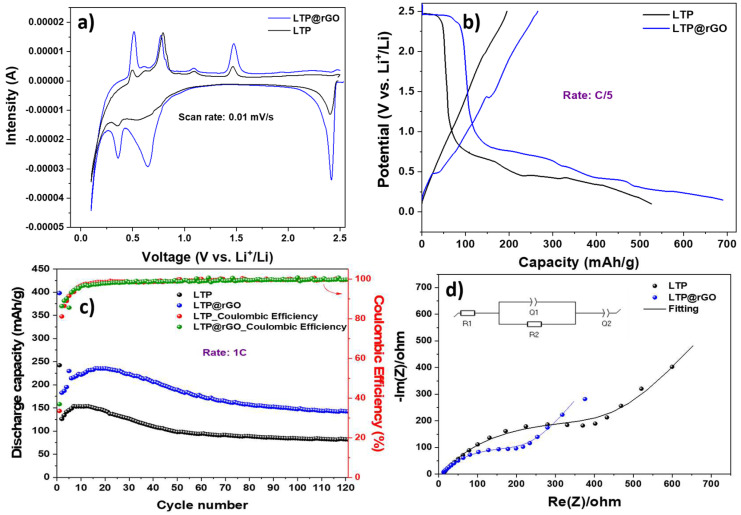
(**a**) Cyclic voltammetry of the initial cycle of LTP and LTP@rGO, (**b**) first galvanostatic discharge/charge curves of LTP and LTP@rGO electrodes at C/5, (**c**) long-term cycling performance of LTP and LTP@rGO electrodes at 1C, (**d**) electrochemical impedance spectra of LTP and LTP@rGO before cycling.

Figure 6a and Figure S11 present a comparison of the cyclic voltammograms for LTP and LTP@rGO measured within the potential range of 0.1–2.5 V at a scan rate of 0.01 mV/s. For the LTP, during the cathodic scan, the peak located at 2.4 V corresponds to the reduction of Ti^4+^ to Ti^3+^ [36], while the peaks located at 0.65 V and 0.35 V indicate a further reduction of Ti^3+^ to Ti^2+^ [37], which allows additional lithium insertion into the LTP crystal lattice, as well as the decomposition of the electrolyte and the formation of the solid electrolyte interface (SEI) layer. These peaks are reversible during the anodic scan at approximately 0.5 V and 0.77 V, suggesting the reversible oxidation of Ti^2+^ back to Ti^3+^. It should be noted that the substantial insertion of lithium at a lower potential results in an irreversible structural change to the material. When it comes to the LTP@rGO electrode, the peak intensities increase, suggesting that rGO improves the prominent peak current, corresponding to the extended voltage plateaus in the charge/discharge curves of LTP@rGO electrodes (Figure 6b) and indicating enhanced redox kinetics. This improvement can be attributed to uniform rGO coating, fine particles, and increased electronic conductivity provided by rGO, which electrically interconnects the LTP particles.

The first galvanostatic discharge/charge profiles of the LTP and LTP@rGO at C/5 rate (1C = 138 mA/g) within the potential window of 0.1–2.5 V are shown in Figure 6b. The first lithiation reached a discharge capacity of 525 mAh/g for the LTP, and achieved 690 mAh/g for LTP@rGO. However, only 37% and 38% of the respective LTP and LTP@rGO initial specific capacities could be reversible in the first charge process. The initial capacity loss is due to the SEI formation as discussed in previous studies [38,39]. The long-term capacity at high cycling rate 1C (Figure 6c) shows that LTP delivers an initial capacity of 242 mAh/g, which reduces to nearly 98 mAh/g after 50 cycles and further declines to 84 mAh/g maintaining a coulombic efficiency of 99.5% after 100 cycles with a capacity retention of 66.5% (considering the second cycle). In contrast, the LTP@rGO demonstrates a significantly higher initial capacity of 398 mAh/g, decreasing to about 189 mAh/g after 50 cycles, maintaining a high capacity of over 147 mAh/g and a coulombic efficiency about 99.5% after 100 cycles with a capacity retention of 80.6%. These results demonstrate the improved lithium ion storage capacity of the prepared LTP@rGO. Nevertheless, when cycling at a low rate of 0.1 C (as depicted in Figure S12), both materials exhibit higher capacities. After 50 cycles, LTP delivers a discharge capacity of 100 mAh/g with a coulombic efficiency of 96%, while LTP@rGO achieves 171 mAh/g with a coulombic efficiency of 98%. Table S3 summarizes the key findings of various NASICON-type-based electrodes. The comparison covers the synthesis method as well as the cell characteristics (such as rate, voltage window, and capacity). In particular, for the same cycling voltage window, the LTP@rGO exhibits performance characteristics that are similar to [40], and in some cases exceed, those reported NASICON electrodes [41,42]. In contrast, the synthesis of LTP@rGO is straightforward and cost-effective involving fewer steps and a lower temperature compared to the complex and multi-step synthesis procedures of the reported materials in Table S3. EIS measurements were performed to verify the predominant role of rGO within the LTP@rGO in enhancing its electrochemical performance from the view of electrical conductivity validation. The Nyquist plots obtained before cycling are shown in Figure 6c. Each Nyquist plot comprises a semicircle at high frequencies and a slop line at low frequencies.

The semicircle observed in the high-frequency region represents the migration of Li^+^ ion from the particle surface layer through the electrolyte. The curved line in the low-frequency region is associated with the diffusion of Li^+^ ions within the electrode. The Z-axis intercept indicates the ohmic resistance R_1_, which encompasses resistance of the separator, electrolyte, and electrode. Using the equivalent circuit model and EC-lab software (version 11.43), EIS curves were fitted as reported previously [43,44]. The double-layer capacitance is represented by a constant phase element (Q_2_). The electrochemical parameters of the equivalent circuit are detailed in Table 2. The R_2_ of the LTP@rGO (209.9 Ω) is smaller than that of the pristine LTP (405.2 Ω), indicating that the appropriate rGO coating facilitates the electron/ion transfer, which may explain the superior electrochemical performance of LTP@rGO.

In addition, as shown in Figure S13, a linear dependence between the peak current Ip and the square root of the scan rate was observed. This relationship allowed for the calculation of the diffusion coefficient of Li^+^ ions in the LTP and LTP@rGO from CV data, using the Randles–Sevcik equation:

$$I_{p}=2.69\times 10^{5}\;n^{3/2}A\;{D_{{Li}^{+}}}^{0.5}\;v^{0.5}\;C$$

where *Ip* represents the peak current (A), D*_Li+_* denotes the Li^+^ diffusion coefficient (cm^2^/s), n is the number of electron transferred in the reaction, A is the electrode’s geometric area of (cm^2^), *v* is the scan rate (V/s), and C is the concentration of the lithium ions in the electrolyte (mol/cm^3^). The values are derived from the slopes of the linear fits for the anodic peak lines of LTP and LTP@rGO. The calculated D values for LTP and LTP@rGO are 3.45 × 10^−11^ cm^2^/s and 1.06 × 10^−10^ cm^2^/s, respectively, at 25 °C for the anodic reaction. The obtained values agree with the enhanced performance of the LTP@rGO.

**Figure 7:**
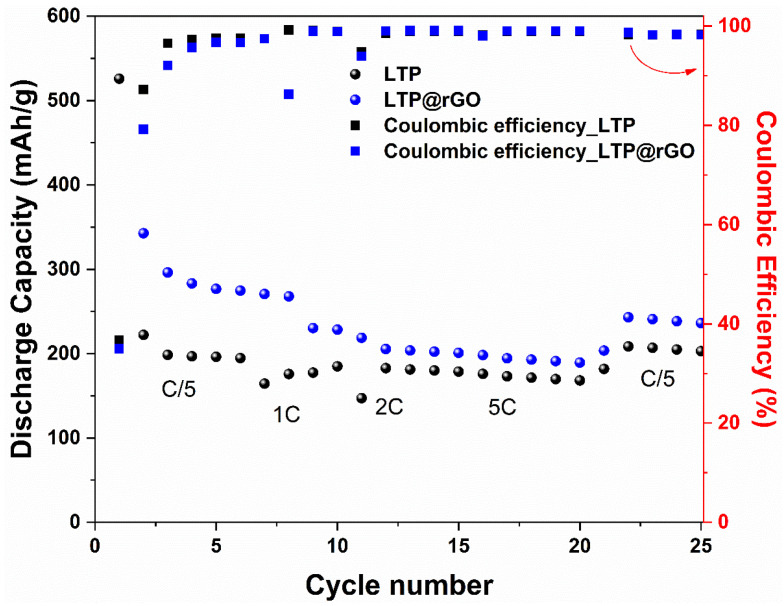
Rate capability of LTP and LTP@rGO electrode.

The cycling performance of LTP and LTP@rGO electrodes was further evaluated at various current rates ranging from C/5 to 5C within the potential window of [0.1 V and 2.5 V]. As shown in Figure 7, LTP@rGO demonstrates improved rate capability. The LTP@rGO delivers discharge capacities of 296, 267, 203, and 192 mAh/g, at the rate of C/5, 1C, 2C, and 5C (Figure S14b), respectively, while LTP shows discharge capacities of 214, 184, 179, and 169 mAh/g, at the rate of C/5, 1C, 2C, and 5C (Figure S14a), respectively. It is possible to state that the improved performance of LTP@rGO is due to the electrical conducting network of rGO as well as the smaller particle and crystallite size. This synergy leads to enhanced performance at a high cycling rate due to the shortened diffusion paths of Li^+^.

**Figure 8:**
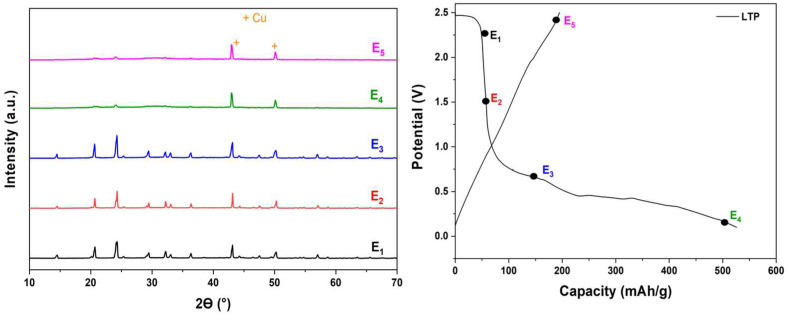
Ex situ XRD spectra of the LTP electrode at various states of discharge and charge within the potential range of [0.1–2.5 V].

It is important to highlight that the initial discharge profile exhibited a significant deviation compared to subsequent profiles, primarily because of the extensive insertion of lithium ions, exceeding the theoretical value of 2 Li^+^, thereby prompting the structural degradation of the pristine LTP phase. The initial capacity of 525 mAh/g for LTP corresponds to an insertion of 7.6 mol of lithium per mole of LTP. The ex situ XRD (Figure 8) at various states of charge reveals that irreversible structural change occurs, resulting in complete amorphization during the initial discharge to 0.1 V. After the initial cycle (Figure S15), the charge–discharge profiles remain nearly identical. Furthermore, the insertion to the Li^+^ ions into the NASICON structure, coupled with the formation of an SEI layer, consumes Li^+^ ions and contributes to the elevated discharge capacity observed in the first cycle. The stability of the structure and the reversibility of the cycling profiles depend on the potential window applied to the material during cycling. Several studies (Table S3), particularly those focusing on LiTi_2_(PO_4_)_3_ material and related compounds, have reported a stable cycling without the structural degradation within the potential window between 1.5 and 3.5 V [45,46]. A study by Srout et al. [40] explored the impact of potential ranges on the Li_1.5_Fe_0.5_Ti_1.5_(PO_4_)_3_ material. Their findings indicate that the Li_1.5_Fe_0.5_Ti_1.5_(PO_4_)_3_ maintains its structural integrity when cycled within a 1.5–3 V potential range. However, cycling within the potential window of 0.5 V to 3 V results in the destruction of the crystalline structure.

## 4. Conclusions

LTP@rGO was successfully prepared through in situ one-step hydrothermal synthesis for the first time at a temperature as low as 250 °C without any further calcination steps. The electrochemical performances of the NASICON materials were investigated, revealing a first discharge capacity of 525 mAh/g for the LTP, and 690 mAh/g for LTP@rGO at a C/5 rate within the potential window of [0.1–2.5 V]. The long-term cycling demonstrated that the LTP@*rGO* achieves 115 mAh/g after 100 cycles at a 1C rate. The TEM shows a good dispersion of LTP particles within the rGO layer, demonstrating the effectiveness of the coating through the one-step hydrothermal procedure. The EIS reveals that the rGO results in improved electron/ion transfer, which may explain the superior electrochemical performance of LTP@rGO.

## Figures and Tables

**Table 1:** Refinement results and crystallite size of the LTP and LTP@rGO.

		LTP	LTP@rGO
**Unit cell** **parameters**	*a* (Å)	8.5032 (1)	8.5113 (4)
	*c* (Å)	20.8351 (1)	20.8547 (5)
	α (°) *= β* (°) = 90, γ (°) = 120		
**Volume (Å^3^)**		1304.63 (2)	1308.37 (9)
**Crystallite size (nm)**		86.6	57.5
**R_B_**		1.29	1.12
**R_p_**		8.47	23.4
**R_wp_**		9.65	22.3

**Table 2:** Impedance parameters of the pristine LTP and LTP@rGO.

Materials	R1 (Ω)	Q1 (Ω⁻¹·cm⁻²·sⁿ), n1	R2 (Ω)	Q2 (Ω⁻¹·cm⁻²·sⁿ), n2	Statistical Parameters
LTP	11.4	3.91× 10^−5^, n1 = 0.72	405.2	0.39 × 10^−3^, n2 = 0.67	χ2 = 0.032
LTP@rGO	9.2	5.4 × 10^−5^, n1 = 0.71	209.9	0.505 × 10^−3^, n2 = 0.69	χ2 = 0.013

## Data Availability

The original contributions presented in this study are included in the article/Supplementary Materials. Further inquiries can be directed to the corresponding authors.

## Supplementary Materials

The following supporting information can be downloaded at: https://www.mdpi.com/article/10.3390/ma18061329/s1, Figure S1: XRD pattern of the synthesized graphene oxide (GO); Figure S2: Raman spectra of graphene oxide (GO); Figure S3: Rietveld refinement patterns of LiTi_2_(PO_4_)_3_ and corresponding crystal structure; Figure S4: XRD of the reduced graphene oxide (rGO) in the hydrothermal autoclave; Figure S5. TEM image of LTP@rGO; Figure S6. TGA curve of rGO; Figure S7. FTIR spectra of GO and rGO; Figure S8. Nitrogen adsorption–desorption isotherms of LTP and LTP@rGO; Figure S9. The average particle size distribution of LTP and LTP@rGO materials; Figure S10. XPS spectra of Ti 2p, P2p, and O 1s of LTP and LTP@rGO materials; Figure S11. Cyclic voltammogams of (a) LTP and (b) LTP@rGO at scan rate of 0.01 mV/s between 0.1 and 2.5 V; Figure S12. Long-term cycling of LTP and LTP@rGO at C/10 rate; Figure S13. Scan rate study between 0.01 and 0.16 mV/s for (a) LTP, (b) LTP@rGO. Corresponding fitted plots of the Randles–Sevcik equation applied to the peak for (c) LTP, (d) LTP@rGO; Figure S14. Galvanostatic charge/discharge profiles of the rate capability experiments of (a) LTP and (b) LTP@rGO; Figure S15. Galvanostatic discharge/charge of the LTP@rGO at C/5 rate; Table S1. Atomic occupation factors, fractional coordinates, isotropic thermal factor and Rietveld refinement results of LTP; Table S2. CHNS analysis of LTP and LTP@rGO materials; Table S3. Electrochemical performances of NASICON electrodes for Li-ion batteries. References [22,23,25,40,41,42,47,48,49,50] are cited in the supplementary materials.
