# Supplementary material for: In-Situ One-Step Hydrothermal Synthesis of LiTi2(PO4)3@rGO Anode for High Performance Lithium-Ion Batteries
Source: Materials (Basel). 2025 Mar 17;18(6):1329. doi: 10.3390/ma18061329 (PMC11943971; doi:10.3390/ma18061329)
Supplement: Supplementary file 1 [file materials-18-01329-s001.zip › materials-3512854-supplementary.pdf]

Supplementary Material

In-Situ One-Step Hydrothermal Synthesis of $LiTi_2(PO_4)_3$@rGO Anode for High Performance Lithium-Ion Batteries

Otmane Zoubir [1,2,*], Abdelfettah Lallaoui [2], M'hamed Oubla [1], Alvaro Y. Tesio [3], Alvaro Caballero [4,*] and Zineb Edfouf [1,2]

[1] Materials and Nanomaterial for Photovoltaics and Electrochemical Storage (MANAPSE), Faculty of Sciences, Mohammed V University in Rabat, Morocco; m.oubla@um5r.ac.ma (M.O.); z.edfouf@um5r.ac.ma (Z.E.)

[2] Moroccan Foundation for Advanced Science Innovation and Research (MAScIR), UM6P, Hay Moulay Rachid, CCI, Ben Guerir 43150, Morocco; a.lallaoui@univ-pau.fr

[3] Centro de Investigación y Desarrollo en Materiales Avanzados y Almacenamiento de Energía de Jujuy (CIDMEJu), Centro de Desarrollo Tecnológico General Manuel Savio, Palpalá 4612, Jujuy, Argentina; atesio@cidmeju.unju.edu.ar

[4] Dpto. Química Inorgánica, Instituto Químico para la Energía y el Medioambiente, Universidad de Córdoba, Campus de Rabanales, 14014 Córdoba, Spain

* Correspondence: otmane.zoubir@um5r.ac.ma (O.Z.); alvaro.caballero@uco.es (A.C.)

Synthesis of graphene oxide

Graphene oxide (GO) was synthesized from graphite powder using a modified Hummer's method [1]. First, 4 g of graphite and 2 g of sodium nitrate ($NaNO_3$) were mixed followed by the addition of 180 mL of sulfuric acid (H_2SO_4) under a regular stirring at 0°C for 2 h. Thereafter 11 g of $KMnO_4$ was gradually added to the solution while keeping the temperature at 25 °C to prevent overheating and explosion. The mixture was diluted by adding 180 mL of distilled water under stirring. Once the temperature reaches 80 °C, 30 mL of 30 % of H_2O_2 was added to ensure the complete reaction with $KMnO_4$. The resulting mixture was washed with distilled water until a pH=7 is reached, followed by centrifugation at 4500 rpm for 30 min and drying at 60 °C for 24 h in air. Finally, the obtained GO was dispersed in distilled water at a concentration of 2 mg/mL.

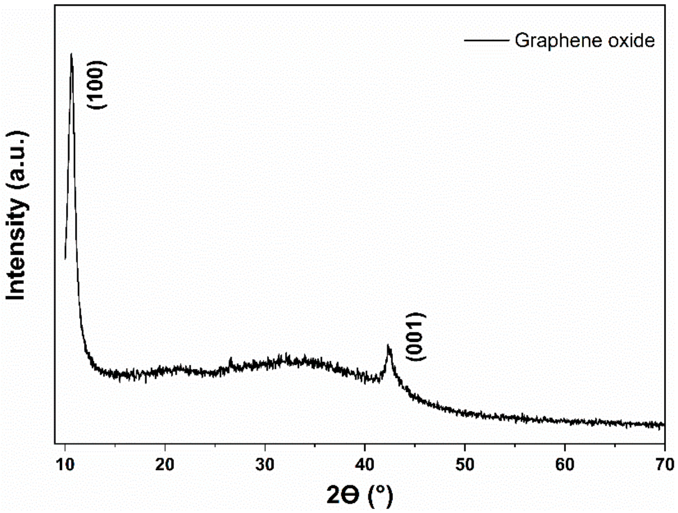

Figure S1. XRD pattern of the synthesized graphene oxide (GO).

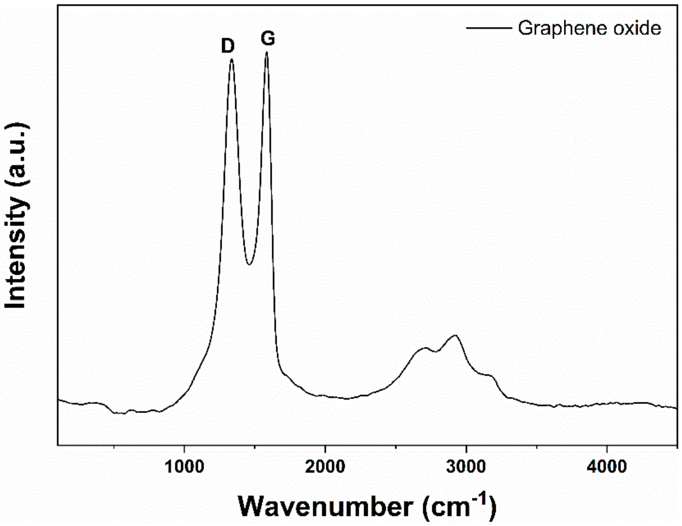

Figure S2. Raman spectra of graphene oxide (GO).

Raman spectra of graphene oxide (**Figure S2**) shows two major peaks, D-band (1338 cm^{-1}) and G band (1568 cm^{-1}). The G-band corresponds to the E_{2g} phonon of the sp^2 C atoms, whereas D band corresponds to the defects and disorder formation [2]. It is worth noting that GO consists of two types of domains: the graphitic domains derived from their parent graphite and highly oxidized domains caused by oxidation. Thus, the D-band provides information related to GO distinct from graphite, particularly oxidation induced defects and disorder.

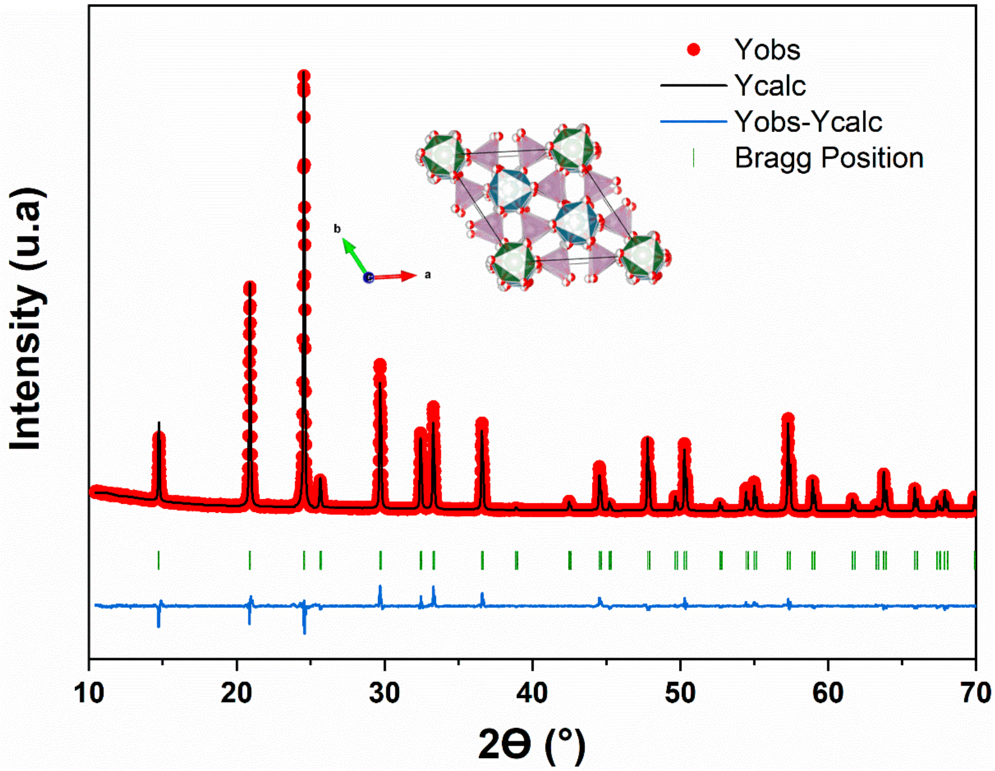

Figure S3. Rietveld refinement patterns of $LiTi_2(PO_4)_3$ and corresponding crystal structure.

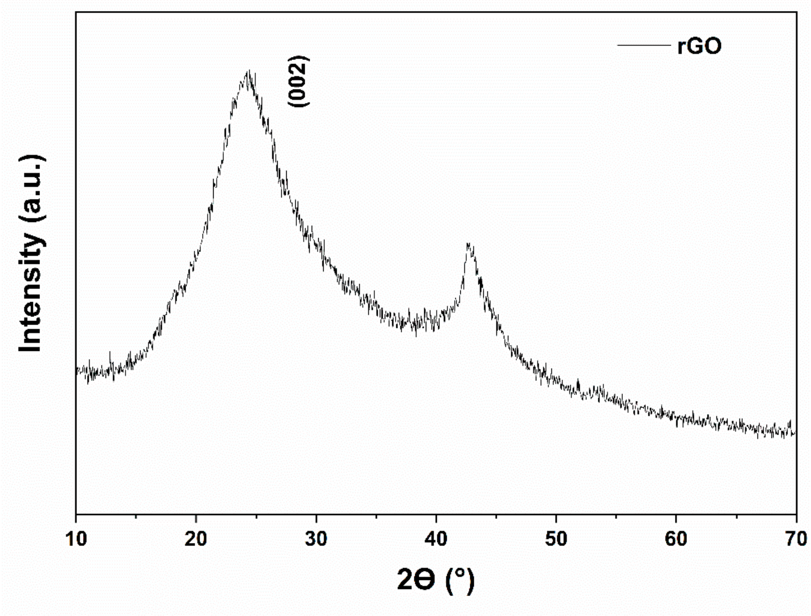

Figure S4. XRD of the reduced graphene oxide (rGO) in the hydrothermal autoclave.

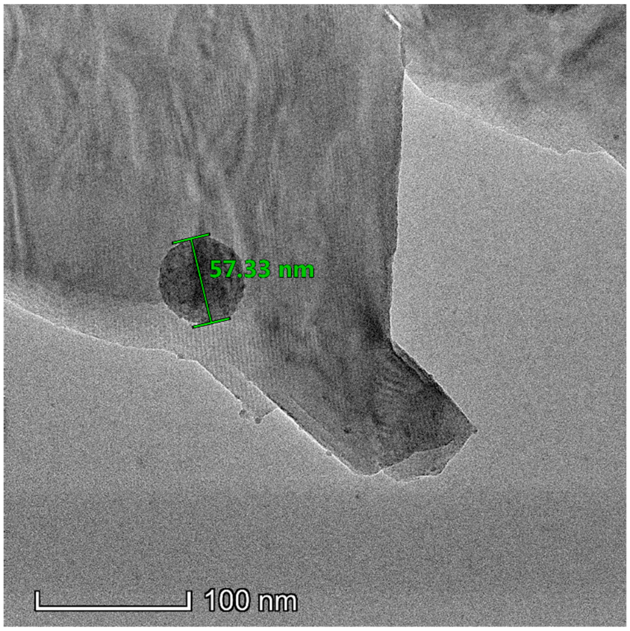

Figure S5. TEM image of LTP@rGO.

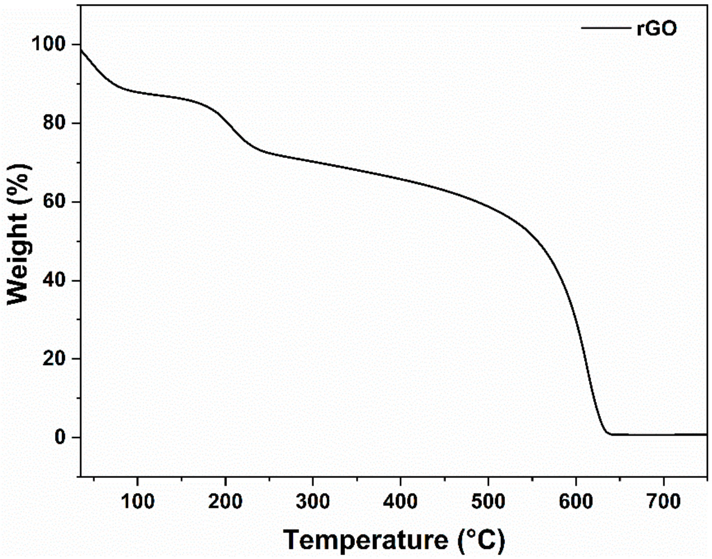

Figure S6. TGA curve of rGO.

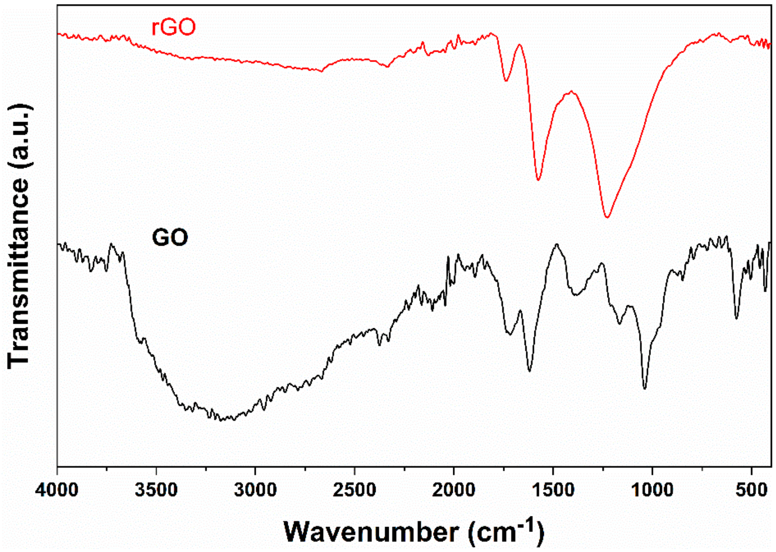

Figure S7. FTIR spectra of GO and rGO.

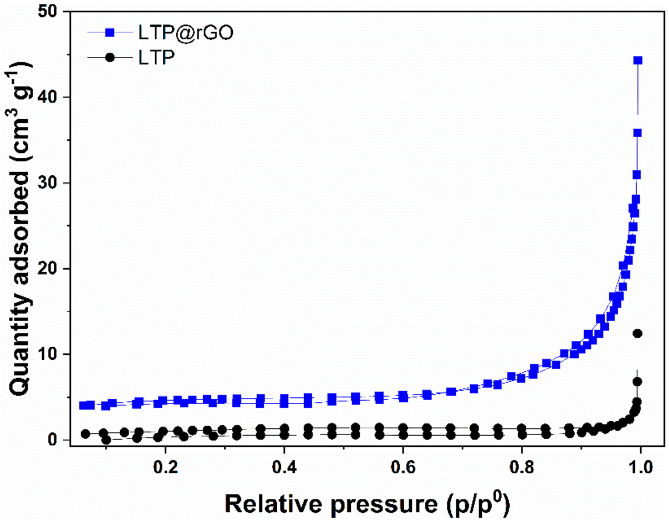

Figure S8. Nitrogen adsorption-desorption isotherms of LTP and LTP@rGO.

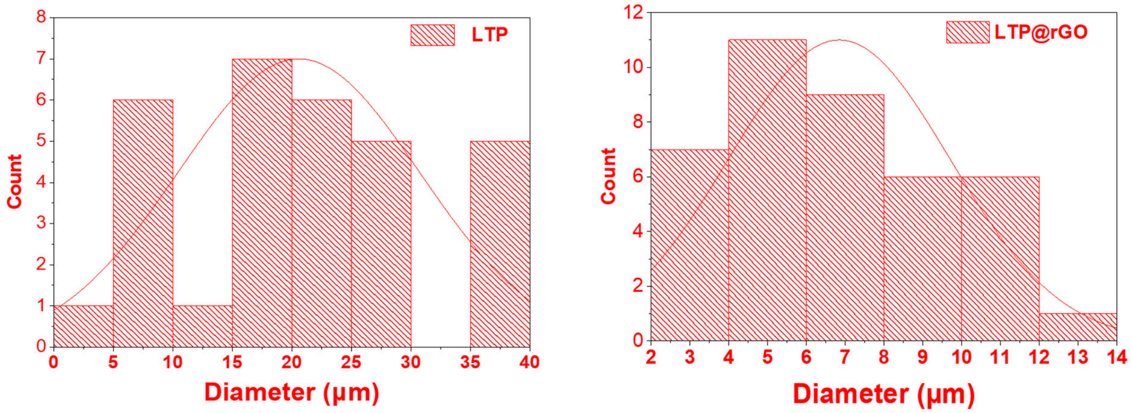

Figure S9. The average particle size distribution of LTP and LTP@rGO materials.

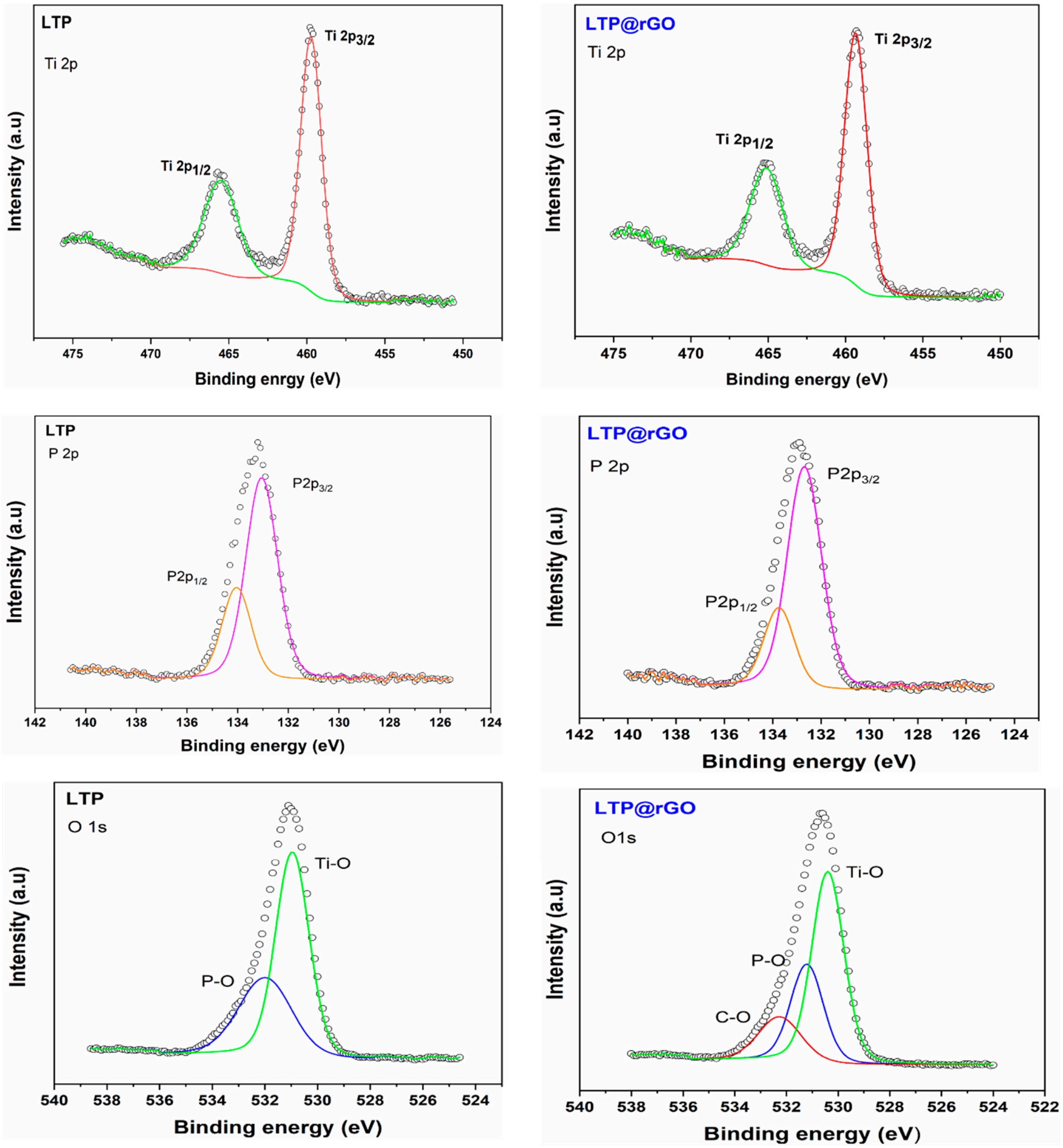

Figure S10. XPS spectra of Ti 2p, P2p, and O 1s of LTP and LTP@rGO materials.

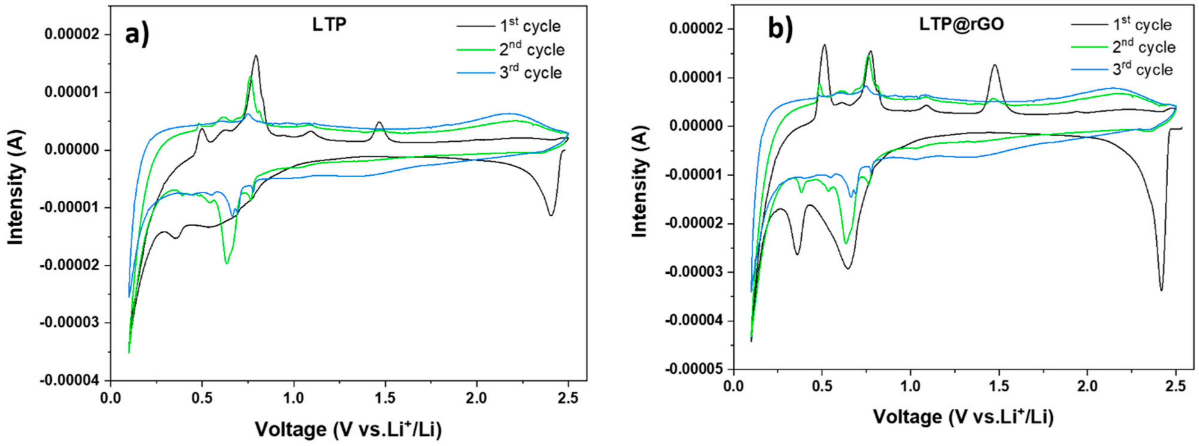

Figure S11. Cyclic voltammogams of (a) LTP and (b) LTP@rGO at scan rate of 0.01 mV/s between 0.1- 2.5 V.

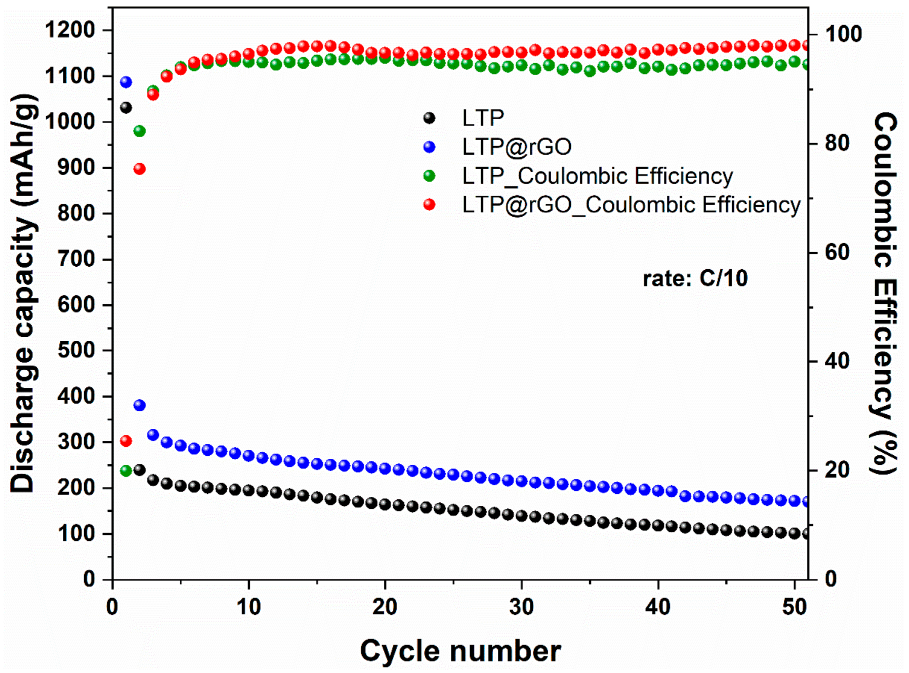

Figure S12. Long term cycling of LTP and LTP@rGO at C/10 rate.

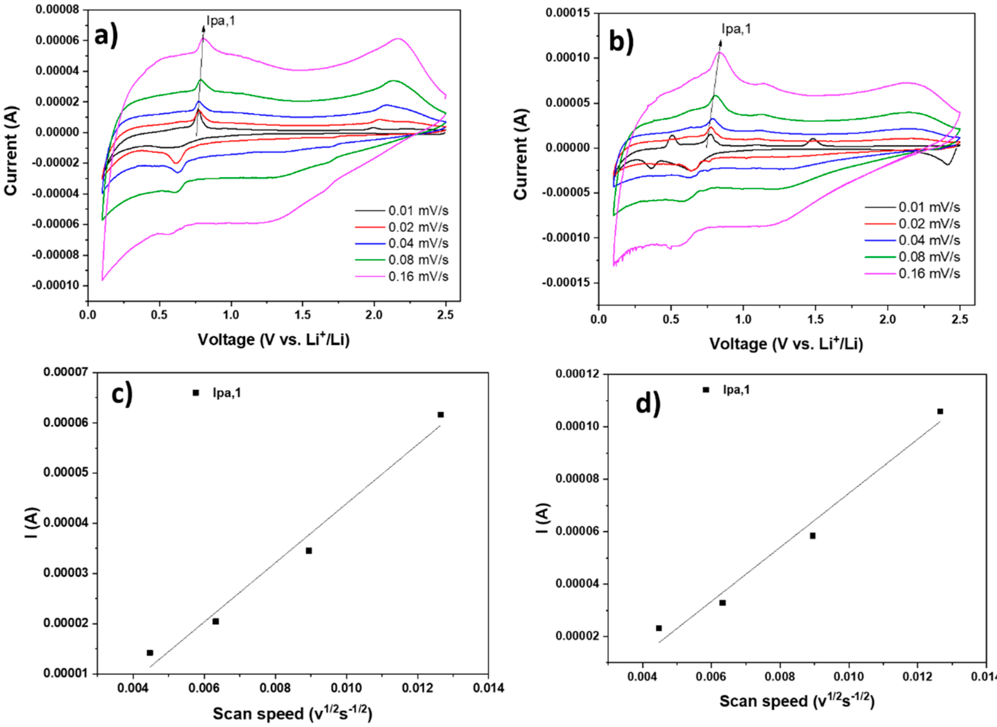

Figure S13. Scan rate study between 0.01 and 0.16 mV/s for (a) LTP, (b) LTP@rGO. Corresponding fitted plots of the Randles-Sevcik equation applied to the peak for (c) LTP, (d) LTP@rGO.

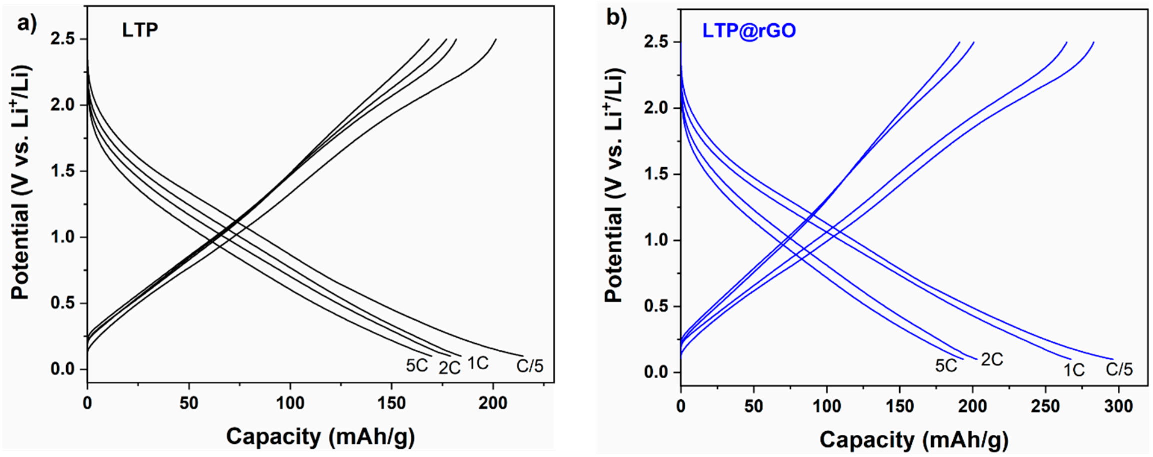

Figure S14. Galvanostatic charge/discharge profiles of the rate capability experiments of (a) LTP and (b) LTP@rGO.

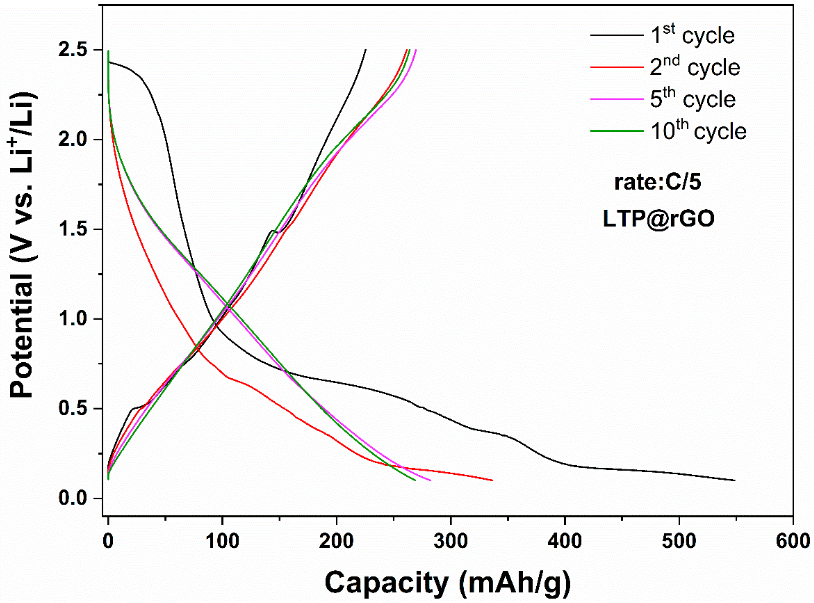

Figure S15. Galvanostatic discharge/charge of the LTP@rGO at C/5 rate.

Table S1. Atomic occupation factors, fractional coordinates, isotropic thermal factor and Rietveld refinement results of LTP

R -3c; Z = 6			$\chi^2 = 5.50$			
$R_F = 2.80$, $R_B = 5.15$				$R_p = 10.7$; $R_{wp} = 8.81$		
Atomic coordinates; (B_{iso}) isotropic displacement parameters (Biso) and Occupancy						
Atom	Wyckoff sites	x/a	y/b	z/c	Biso (Å^2)	Occupancy
Li	6b	0.00000	0.00000	0.00000	4.5384	0.1600
Ti	12c	0.00000	0.00000	0.1414 (1)	1.2341	0.3000
P	18e	0.2908 (1)	0.00000	0.2500	2.5495	0.5000
O1	36f	0.1809 (4)	0.9866 (4)	0.1888 (1)	3.1016	1.0000
O2	36f	0.1855 (3)	0.1583(3)	0.0783 (2)	2.14486	1.0000

Table S2. CHNS analysis of LTP and LTP@rGO materials.

Compound	(N) %	(C) %
LTP	0.000	N.D.*
LTP@rGO	0.000	1.316

Table S3. Electrochemical performances of NASICON electrodes for Li-ion batteries.

Material	Preparation Method	Voltage window / V	Final capacity/mAh/g (Cycle)	Rate	Ref
$LiTi_2(PO_4)_3/C$	ball milling (400 rpm for 3 h)	1.5-3	140.7 (100)	0.1C	[3]
$LiTi_2(PO_4)_3$@rGO	microwave-assisted one-pot method	2 –3	98.76 (100)	1C	[4]
$Li_{1.5}Fe_{0.5}Ti_{1.5}(PO_4)_3/C$	sol-gel reaction	1.5–3.0<br>0.5–3.0	141 (first)<br>567 (first)	0.1C	[5]
$LiTi_2(PO_4)_3/C$	Solvothermal method	1.5–3.5	107.6 (50)	1C	[6]
$LiTi_2(PO_4)_3$	Solid state reaction	0–2.5	360 (first)	0.1 mA cm^{-2}	[7]
mesoporous LATP	Hydrothermal	2.5 – 3.5<br><br>0.05 – 3	71 (10)<br><br>122 (50)	0.1C	[8]
$LiTi_2(PO_4)_3$	Solvothermal	1.5 – 3.5	64 (1000)	10 C	[9]
$LiTi_2(PO_4)_3/C$	co-precipitation	1.5 – 3.5	116.9 (1000)	5 C	[10]

References

1. Zaaba, N.I.; Foo, K.L.; Hashim, U.; Tan, S.J.; Liu, W.-W.; Voon, C.H. Synthesis of Graphene Oxide Using Modified Hummers Method: Solvent Influence. *Procedia engineering* **2017**, *184*, 469–477.
2. Khan, Q.A.; Shaur, A.; Khan, T.A.; Joya, Y.F.; Awan, M.S. Characterization of Reduced Graphene Oxide Produced through a Modified Hoffman Method. *Cogent Chemistry* **2017**, *3*, 1298980.
3. Liu, L.; Zhou, M.; Wang, G.; Guo, H.; Tian, F.; Wang, X. Synthesis and Characterization of $LiTi_2(PO_4)_3/C$ Nanocomposite as Lithium Intercalation Electrode Materials. *Electrochimica acta* **2012**, *70*, 136–141.
4. Roh, H.-K.; Kim, H.-K.; Roh, K.C.; Kim, K.-B. $LiTi_2(PO_4)_3$/Reduced Graphene Oxide Nanocomposite with Enhanced Electrochemical Performance for Lithium-Ion Batteries. *RSC advances* **2014**, *4*, 31672–31677.
5. Srout, M.; Lasri, K.; Dahbi, M.; Kara, A.; Tetard, L.; Saadoune, I. Understanding of the Li-Insertion Process in a Phosphate Based Electrode Material for Lithium Ion Batteries. *Journal of Power Sources* **2019**, *435*, 226803.
6. Yu, S.; Tempel, H.; Schierholz, R.; Aslanbas, Ö.; Gao, X.; Mertens, J.; de Haart, L.G.; Kungl, H.; Eichel, R.-A. $LiTi_2(PO_4)_3/C$ Anode Material with a Spindle-Like Morphology for Batteries with High Rate Capability and Improved Cycle Life. *ChemElectroChem* **2016**, *3*, 1157–1169.
7. Wang, G.X.; Bradhurst, D.H.; Dou, S.X.; Liu, H.K. $LiTi_2(PO_4)_3$ with NASICON-Type Structure as Lithium-Storage Materials. *Journal of power sources* **2003**, *124*, 231–236.
8. Bhanja, P.; Senthil, C.; Patra, A.K.; Sasidharan, M.; Bhaumik, A. NASICON Type Ordered Mesoporous Lithium-Aluminum-Titanium-Phosphate as Electrode Materials for Lithium-Ion Batteries. *Microporous and Mesoporous Materials* **2017**, *240*, 57–64.
9. Guo, Z.; Qin, X.; Xie, Y.; Lei, C.; Wei, T.; Zhang, Y. Advanced NASICON-Type $LiTi_2(PO_4)_3$ as Electrode Materials for Lithium-Ion Batteries. *Chemical Physics Letters* **2022**, *806*, 140010.
10. Sun, J.; Sun, Y.; Gai, L.; Jiang, H.; Tian, Y. Carbon-Coated Mesoporous $LiTi_2(PO_4)_3$ Nanocrystals with Superior Performance for Lithium-Ion Batteries. *Electrochimica Acta* **2016**, *200*, 66–74.
